# Reference ranges for ambulatory heart rate measurements in a middle-aged population

**DOI:** 10.1136/heartjnl-2023-323681

**Published:** 2024-04-05

**Authors:** Anders Paul Persson, Alexandra Måneheim, Johan Economou Lundeberg, Artur Fedorowski, Jeff S Healey, Johan Sundström, Gunnar Engström, Linda S B Johnson

**Affiliations:** 1 Department of Clinical Sciences, Lund University, Malmö, Sweden; 2 Department of Clinical Physiology, Skånes universitetssjukhus Malmö, Malmö, Sweden; 3 Department of Clinical Physiology, Skånes universitetssjukhus Lund, Lund, Sweden; 4 Department of Clinical Sciences, Lund University Faculty of Medicine, Malmö, Sweden; 5 Department of Medicine, Karolinska Institute, Solna, Sweden; 6 Population Health Research Institute, Hamilton, Ontario, Canada; 7 Department of Medicine, McMaster University, Hamilton, Ontario, Canada; 8 Department of Medical Sciences, Uppsala University, Uppsala, Sweden; 9 The George Institute for Global Health, Newtown, New South Wales, Australia

**Keywords:** Bradycardia, Electrocardiography, Epidemiology

## Abstract

**Background:**

Elevated heart rate (HR) predicts cardiovascular disease and mortality, but there are no established normal limits for ambulatory HR. We used data from the Swedish CArdioPulmonary Imaging Study to determine reference ranges for ambulatory HR in a middle-aged population. We also studied clinical correlates of ambulatory HR.

**Methods:**

A 24-hour ECG was registered in 5809 atrial fibrillation-free individuals, aged 50–65 years. A healthy subset (n=3942) was used to establish reference values (excluding persons with beta-blockers, cardiovascular disease, hypertension, heart failure, anaemia, diabetes, sleep apnoea or chronic obstructive pulmonary disease).

Minimum HR was defined as the lowest 1-minute HR. Reference ranges are reported as means±SDs and 2.5th–97.5th percentiles. Clinical correlates of ambulatory HR were analysed with multivariable linear regression.

**Results:**

The average mean and minimum HRs were 73±9 and 48±7 beats per minute (bpm) in men and 76±8 and 51±7 bpm in women; the reference range for mean ambulatory HR was 57–90 bpm in men and 61–92 bpm in women. Average daytime and night-time HRs are also reported. Clinical correlates, including age, sex, height, body mass index, physical activity, smoking, alcohol intake, diabetes, hypertension, haemoglobin level, use of beta-blockers, estimated glomerular filtration rate, per cent of predicted forced expiratory volume in 1 s and coronary artery calcium score, explained <15% of the interindividual differences in HR.

**Conclusion:**

Ambulatory HR varies widely in healthy middle-aged individuals, a finding with relevance for the management of patients with a perception of tachycardia. Differences in ambulatory HR between individuals are largely independent of common clinical correlates.

**WHAT IS ALREADY KNOWN ON THIS TOPIC**

- Ambulatory heart rate varies widely and is only explained by clinical correlates to a minor degree.

**WHAT THIS STUDY ADDS**

- This study presents reference ranges of ambulatory heart rate in a middle-aged population.

**HOW THIS STUDY MIGHT AFFECT RESEARCH, PRACTICE OR POLICY**

- These normal limits for heart rate can be used for the interpretation of ambulatory ECGs, for instance, to determine whether patients with subjective perception of tachycardia have abnormally elevated heart rates.

## Introduction

High resting heart rates have been linked to cardiovascular, cancer and all-cause death.^1^ The finding has been consistent in population-based studies,^2–4^ as well as in studies of individuals with hypertension,^5^ diabetes,^6^ chronic obstructive pulmonary disease,^7^ cardiovascular disease^8^ and heart failure.^9^ Low heart rate, on the other hand, is associated with atrial fibrillation.^10–12^ Regardless of whether these associations imply a causal relation between heart rate and outcomes or not, heart rate measurements can be useful for disease prediction. Ambulatory heart rates can be inexpensively, reliably and non-invasively measured, and ambulatory ECG monitoring is frequently performed in clinical practice. Furthermore, patients with post-COVID-19 condition frequently report inappropriately elevated resting heart rates,^13 14^ a symptom which cannot be put in context without knowledge of normal limits. Despite this, studies that report reference ranges and or predictors of heart rate at 24-hour ECG (24hECG) are lacking.

We aimed to describe reference ranges for, and predictors of ambulatory heart rate measured with 24hECG in a middle-aged general population sample.

## Methods

### Study sample

**Figure 1:**
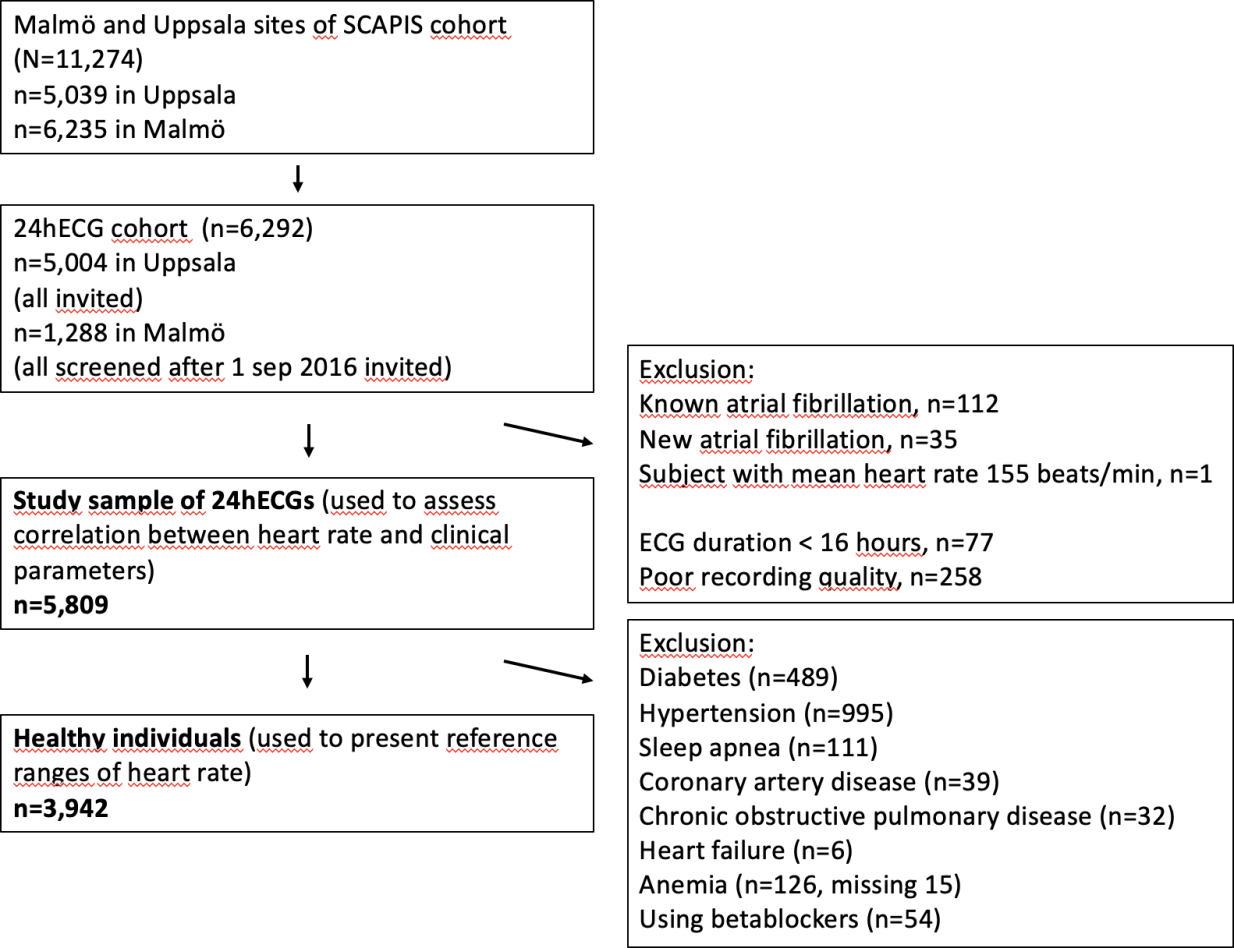
Derivation of study population. 24hECG, 24-hour ECG; SCAPIS, Swedish CArdioPulmonary Imaging Study.

The population-based Swedish CArdioPulmonary Imaging Study (SCAPIS) cohort included 30 154 participants aged 50–65 years old recruited from the general population, in six municipal centres containing university hospitals in Sweden (Gothenburg, Linköping, Malmö, Stockholm, Uppsala and Umeå). Examination with 24hECG was part of the protocol at the Malmö (6235 participants) and Uppsala (5038 participants) sites. All participants examined in Malmö between September 2016 and 2018 as well as all participants in Uppsala were asked to contribute a 24hECG registration, of which 1288 (Malmö) and 5004 (Uppsala) accepted. After excluding individuals with a previous diagnosis of atrial fibrillation (n=112), atrial fibrillation during the ambulatory ECG recording (n=35), registration duration <16 hours (n=77) or insufficient 24hECG registration quality (n=258), and one participant with an implausible mean heart rate (155 beats per minute (bpm)), 5809 participants constituted the final study population (figure 1). Reference ranges for heart rate were reported in a healthy population subset after exclusion of participants with known prevalent cardiovascular diseases, hypertension, heart failure, anaemia, diabetes, obstructive sleep apnoea, chronic obstructive pulmonary disease, and individuals using beta-blockers, resulting in a healthy reference cohort of 3942 subjects. Neither the study participants nor the public were involved in the design, conduct, reporting or dissemination plans of this study.

### Data collection

24hECGs were recorded using CardioSpy equipment (Labtech, Debrecen, Hungary), with X, Y, Z coupling and sampling frequency of 256 Hz, and measures of heart rate were derived from the CardioSpy ECG analysis software. Cases of atrial fibrillation during 24hECG were detected using a Food and Drug Association and CE-approved artificial intelligence algorithm (MEDICALgorithmics, Warsaw, Poland). The measures of heart rate included in this study were mean heart rate during the entire recording, minimum heart rate during 1 min, and mean day (06:00–22:00) and night-time (22:00–06:00) heart rates. Nightly dip in heart rate was calculated as daytime heart rate minus night-time heart rate. Maximum heart rate during 1 min was not studied in detail due to its dependence on maximum exertion but percentiles are presented in online supplemental table 1.

10.1136/heartjnl-2023-323681.supp1 Supplementary data

Each participant completed an extensive health and lifestyle questionnaire from which data concerning physical activity, smoking and alcohol use were obtained, as was the prevalence of known prevalent diseases and drug treatment. A capillary glucose sample was collected, and if elevated (≥7 mmol/L), a repeat measurement was performed on another day to diagnose diabetes.

Height was measured to the nearest centimetre and weight was measured on a digital scale in light indoor clothing without shoes. Body mass index (BMI) was calculated as kg/m^2^ and stratified at 25 kg/m^2^, 25–30 kg/m^2^ and >30 kg/m^2^. Dynamic spirometry (Jaeger MasterScreen PFT; Carefusion, Hoechberg, Germany) was performed 15 min after bronchodilation using 400 µg salbutamol with subjects in the sitting position and wearing a nose clip, and forced expiratory volume during 1 s (FEV_1_) was measured according to the American Thoracic Society and European Respiratory Society standards.^15 16^ The Hedenström formula was used to calculate per cent of predicted FEV_1_ (FEV_1_%predicted).^17 18^ Smoking status was categorised as current, former or never smoker. Reported leisure time physical activity was categorised as low (mostly sedentary or some light physical activity) or high (physical activity with moderate to strenuous intensity at least 2 hours per week). Alcohol intake in units per week was calculated by multiplying average times per week of drinking during the last year with the typical intake on a day of drinking. Alcohol intake was then stratified into two groups (above or below the median intake of 2.6 units per week).

A fasting venous blood sample was retrieved and haemoglobin and creatinine concentrations were measured using standard laboratory procedures at the Uppsala and Malmö University Hospitals. Estimated glomerular filtration rate (eGFR) was calculated using the creatinine-based Chronic Kidney Disease Epidemiology Collaboration formula.^19^

CT was performed in all participants using Siemens Definition Flash 2×128 slice, stellar detector, 4D‐Care dose, Care‐kV and sinogram‐affirmed iterative reconstruction (Forchheim, Germany). Coronary artery calcium score was calculated using the Agatston score^20^ and stratified into three groups: 0, 1–99 and ≥100.

### Statistical analyses

Reference ranges for heart rates are reported as means (±SD and percentiles) and presented in histograms and sex-specific cumulative distribution function plots. The reported percentiles were taken directly from the observed distribution and not derived from the SD. The sex-specific cumulative distribution function plots for average day and night-time heart rate are presented in online supplemental figure 1. Multivariable linear regression models were used to analyse the association between heart rate and a prespecified set of predictors including age, sex, height, BMI, physical activity, smoking, alcohol intake, diabetes, hypertension, haemoglobin level, use of beta-blockers, eGFR, FEV_1_%predicted and coronary artery calcium score. All continuous parameters were assessed visually in histograms for normality. Collinearity was ruled out using the variance inflation factor, using a cut-off of 2.5. Model diagnostics were performed using plots of residuals and fitted values and histograms of the residuals. No violations of the assumptions were observed (online supplemental figures 2–5). Linear regression robust to heteroskedasticity was also performed for mean and minimum heart rate with virtually unchanged results (online supplemental table 3).

All statistical analyses were performed using Stata V.15.1 (StataCorp, College Station, Texas, USA).

## Results

The age of study participants was evenly distributed between 50 and 65 years of age and 52% were women. While 22% had hypertension, heart failure was rare (0.3%). Study sample characteristics are presented in more detail in table 1.

**Table 1:** Sample characteristics

	All (N=5809)	Women	Men	Healthy reference sample (n=3942)
Age, mean (range)	58 (50–65)	58 (50–65)	58 (50–65)	57 (50–65)
Women, %	53	–	–	54
Height, cm (SD)	172 (9.7)	166 (6.5)	179 (7.0)	172 (9.7)
BMI, %				
<25	35	42	27	40
25–30	44	38	51	44
>30	21	20	22	16
Smoking, %				
Never	56	53	59	58
Former	34	37	30	32
Current	11	11	11	10
Alcohol intake units/week, median (IQR)	2.6 (1.1–4.1)	1.1 (0.4–3.8)	2.6 (1.1–6.0)	2.6 (1.1–4.1)
Physical activity, %				
Low	57	60	54	54
High	43	40	46	46
Diabetes, %	25	7	11	–
Hypertension, %	21	20	22	–
Use of oral beta-blockers, %	5,8	6.1	5.5	–
FEV_1_%predicted (SD)	109 (15)	109 (15)	108 (14)	110 (14)
Coronary artery calcium score, %				
0	61	74	46	67
1–99	27	21	35	25
≥100	11	5	19	8
eGFR, mL/min/1.73 m^2^ (IQR)	88 (79–96)	88 (78–96)	89 (80–96)	88 (79–96)
Haemoglobin, g/L (SD)	142 (12)	135 (9.1)	149 (9.4)	142 (11)
Sleep apnoea, %	3.7	2.6	4.9	–
Coronary artery disease, %	2.0	1.1	3.0	–
Heart failure, %	0.3	0.3	0.4	–

BMI, body mass index; eGFR, estimated glomerular filtration rate; FEV_1_%predicted, per cent of predicted forced expiratory volume during 1 s.

### Reference ranges for heart rate

A healthy population subset was used to determine reference ranges for heart rate measures. The mean heart rate was 73 (±9) bpm on average for men and 76 (±8) bpm on average for women, and the minimum heart rate was 48 (±7) bpm on average for men and 51 (±7) bpm on average for women.

**Figure 2:**
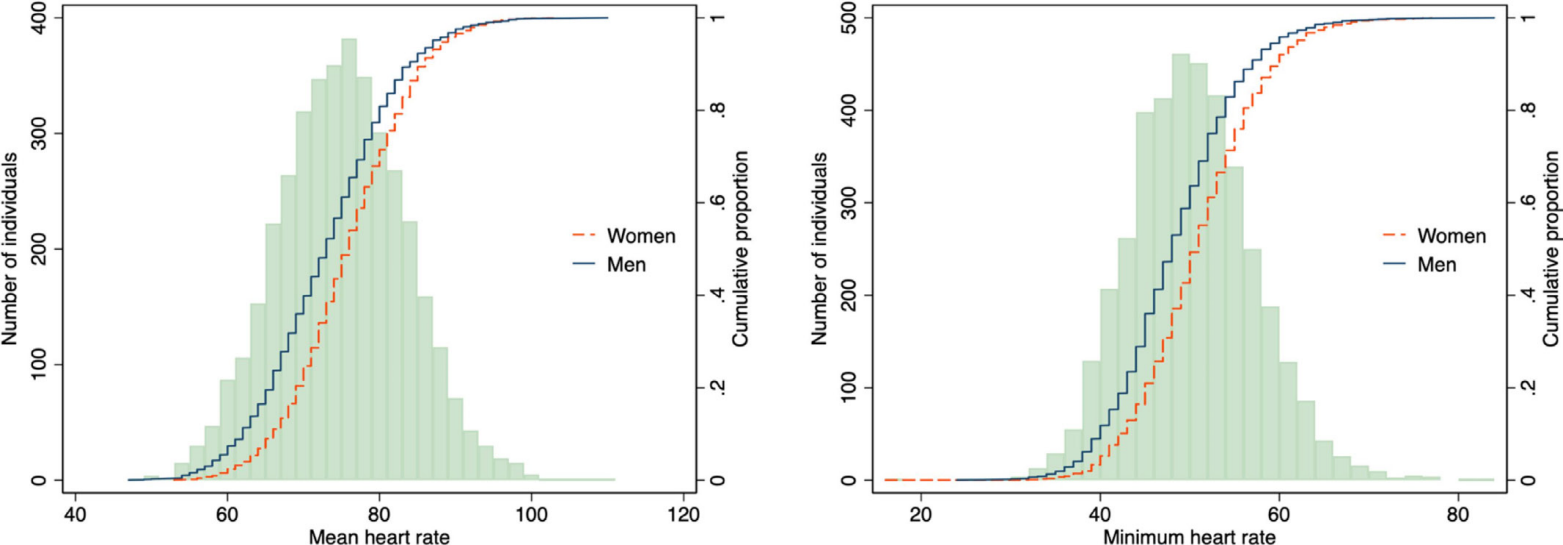
Heart rates in the healthy reference sample, histogram and sex-specific cumulative distribution curves.

The range of heart rate was wide; the 2.5th–97.5th percentile of mean heart was 57–90 bpm in men and 61–92 bpm in women (table 2 and figure 2). Reference heart rates (only including healthy individuals) did not differ substantially from heart rates in the entire study population (online supplemental table 2). The average nightly dip in heart rate was 15 bpm in men and 16 bpm in women. Heart rate parameters are reported in detail for both men and women in table 2.

**Table 2:** Reference ranges for measures of ambulatory heart rate

	Mean heart rate (beats/min)		Minimum heart rate* (beats/min)		Average daytime heart rate (beats/min)		Average night-time heart rate (beats/min)	
	Men	Women	Men	Women	Men	Women	Men	Women
Healthy individuals (1825 men and 2117 women)†								
Mean (SD)	73 (9)	76 (8)	48 (7)	51 (7)	78 (9)	81 (8)	63 (9)	65 (8)
Percentiles								
**2.5**	**57**	**61**	**36**	**39**	**60**	**65**	**48**	**51**
5	59	63	38	40	63	67	50	53
25	67	71	44	46	71	75	57	60
** 50**	**73**	**76**	**48**	**51**	**78**	**81**	**63**	**65**
75	79	81	53	55	84	86	68	71
95	87	89	60	62	93	94	79	80
**97.5**	**90**	**92**	**63**	**65**	**96**	**97**	**83**	**83**

*Lowest average heart rate during 1 min.

†Based on 3942 (2117 women) healthy individuals, excluding individuals with new or known diabetes (490) or known prevalent hypertension (n=995), sleep apnoea (n=111), coronary artery disease (n=39), chronic obstructive pulmonary disease (n=32), heart failure (n=6) or anaemia (haemoglobin <120 g/L in women and <130 g/L in men) (n=126, missing 15) and individuals using beta-blockers (54).

### Clinical predictors of heart rate

Clinical predictors of heart rate are calculated in the full study sample and presented in table 3. We included 14 clinical variables in the multivariable linear regression, and 11 (sex, height, BMI, smoking, high physical activity, alcohol intake, diabetes, using oral beta-blockers, FEV_1_%predicted, eGFR and haemoglobin) were statistically significantly associated with mean heart rate (table 3). The associations were overall rather weak, however. For example, the mean heart rate was on average only 4.5 bpm lower in beta-blocker users and 2.7 bpm higher in smokers, after multivariable adjustment (table 3). In keeping with this, the variation in heart rate was explained by the clinical variables included in the multivariable model only to a small degree; adjusted R^2^ and unadjusted R^2^ in the multivariable model were <15% and <16%, respectively, for all the studied measures of heart rate. In sex-stratified multivariable models, the adjusted R^2^ for mean heart rate was 16% in men and 10% in women. Within the age range of this study, heart rate was virtually unchanged with age.

**Table 3:** Multivariable linear regression models for ambulatory heart rate measures

N=5021	Mean heart rate, beats/min (95% CI)		Minimum heart rate, beats/min (95% CI)		Average daytime heart rate, beats/min (95% CI)		Average night-time heart rate, beats/min (95% CI)	
	Beta (95% CI)	t ratio*	Beta (95% CI)	t ratio*	Beta (95% CI)	t ratio*	Beta (95% CI)	t ratio*
Age (per 1 year)	−0.0 (−0.1, 0.0)	−1.4	0.1 (0.0, 0.1)	3.3	−0.0 (−0.1, 0.0)	−1.4	−0.0 (−0.1, 0.0)	−0.6
Men (vs women)	**−2.8** **(−3.5, −2.0)**	−7.3	**−2.2** **(−2.9, −1.6)**	−7.1	**−3.2** **(−4.0, −2.4)**	−7.7	**−2.0** **(−2.8, −1.3)**	−5.2
Height (per 10 cm)	**−0.8** **(−1.2, −0.5)**	−4.9	**−0.8** **(−1.0, −0.5)**	−5.2	**−0.8** **(−1.2, −0.5)**	−4.4	**−0.9** **(−1.2, −0.6)**	−5.1
Body mass index, kg/m^2^								
<25	Ref	Ref	Ref	Ref	Ref	Ref	Ref	Ref
25–30	**0.9** **(0.4, 1.4)**	3.5	**0.8** **(0.4, 1.3)**	3.8	**0.6** **(0.1, 1.2)**	2.3	**1.4** **(0.9, 2.0)**	5.4
>30	**1.9** **(1.2, 2.6)**	5.6	**1.4** **(0.9, 2.0)**	5.1	**1.4** **(0.7, 2.1)**	3.9	**2.9** **(2.3, 3.6)**	8.5
Smoking								
Never	Ref	Ref	Ref	Ref	Ref	Ref	Ref	Ref
Former	**1.2** **(0.7, 1.7)**	4.9	**0.9** **(0.5, 1.3)**	4.3	**1.1** **(0.6, 1.7)**	4.2	**1.3** **(0.8, 1.9)**	5.2
Current	**2.7** **(1.9, 3.4)**	6.7	**2.2** **(1.6, 2.9)**	6.8	**2.2** **(1.3, 3.0)**	5.0	**3.7** **(2.9, 4.5)**	9.2
High physical activity (vs low)	**−3.6** **(−4.1, −3.1)**	−15.1	**−2.7** **(−3.1, −2.3)**	−13.5	**−3.7** **(−4.2, −3.2)**	−14.4	**−3.3** **(−3.8, −2.9)**	−13.7
Alcohol intake, above median (vs below median)	**0.7** **(0.2, 1.1)**	3.0	**0.8** **(0.4, 1.2)**	4.1	0.5 (0.0, 1.0)	2.1	**1.0** **(0.6, 1.5)**	4.3
Diabetes (yes vs no)	**2.0** **(1.1, 2.8)**	4.5	**2.1** **(1.4, 2.8)**	5.8	**1.7** **(0.8, 2.6)**	3.6	**2.5** **(1.6, 3.4)**	5.5
Hypertension (yes vs no)	0.4 (−0.1, 1.0)	1.5	0.1 (−0.4, 0.6)	0.4	0.5 (−0.1, 1.2)	1.6	0.3 (−0.3, 0.9)	0.8
Using oral beta-blockers (yes vs no)	**−4.5** **(−5.5, −3.4)**	−8.5	**−0.9** **(−1.8, −0.1)**	−2.2	**−5.7** **(−6.8, −4.5)**	−9.9	**−1.8** **(−2.8, −0.7)**	−3.3
FEV_1_%predicted, per 10% increase	**−0.3** **(−0.4, −0.1)**	−3.6	**−0.3** **(−0.5, −0.2)**	−4.8	−0.2 (−0.4, 0.0)	−2.4	**−0.4** **(−0.6, −0.3)**	−5.3
Coronary artery calcium score								
0	Ref	Ref	Ref	Ref	Ref	Ref	Ref	Ref
1–99	0.4 (−0.1, 1.0)	1.6	0.4 (−0.1, 0.8)	1.6	0.4 (−0.2, 1.0)	1.3	0.5 (0.0, 1.1)	1.8
≥100	0.6 (−0.2, 1.4)	1.5	0.5 (−0.1, 1.2)	1.6	0.5 (−0.4, 1.3)	1.1	0.8 (0.0, 1.6)	2.0
eGFR (per 10 mL/min/1.73 m^2^)	**0.6** **(0.4, 0.8)**	6.1	**0.4** **(0.3, 0.6)**	5.0	**0.7** **(0.5, 0.9)**	6.2	**0.5** **(0.3, 0.7)**	5.1
Haemoglobin (per 10 g/L increase)	**0.7** **(0.5, 1.0)**	5.7	**0.3** **(0.1, 0.5)**	2.5	**0.8** **(0.6, 1.1)**	6.1	**0.4** **(0.2, 0.7)**	3.5
Constant†	76.1 (75.4, 76.8)		49.9 (49.3, 50.5)		81.5 (80.7, 82.3)		64.4 (63.7, 65.2)	
Unadjusted R^2^	0.149		0.143		0.133		0.151	
Adjusted R^2^	0.146		0.140		0.130		0.148	

Bold numbers indicate statistical significance (p<0.05).

*Larger t ratios (both positive and negative) imply larger impacts on heart rate.

†The constant is what is expected for an individual with values that have been set as reference as stated in the table and age 50 years old, the mean of the continuous variables height (172 cm), eGFR (89 mL/min/1.73 m^2^), haemoglobin (142 g/L) and FEV_1_%predicted (109%).

eGFR, estimated glomerular filtration rate; FEV_1_%predicted, per cent of predicted forced expiratory volume during 1 s.

## Discussion

This is the largest population-based study of heart rate at ambulatory ECG to date, and the first to provide reference ranges for measures of heart rate at 24hECG in a middle-aged population. We found the interindividual differences in heart rate to be large and largely independent of clinical risk factors for cardiovascular disease.

Previous studies have shown that there is prognostic value in ambulatory heart rate measurements,^21^ but since reference ranges have been lacking, it has not been clear how ambulatory heart rate measurements could be used in clinical practice. This matter has become even more relevant in the context of the COVID-19 pandemic and the emergence of post-COVID-19 syndromes, where a feeling of increased heart rate is often reported, but objective guidelines as to what constitutes elevated heart rate and pre-COVID-19 references for ambulatory heart rate have been lacking. For instance, although inappropriate sinus tachycardia is typically diagnosed when average heart rate on 24hECG monitoring exceeds 90 bpm,^22^ population-based studies supporting this diagnostic threshold are limited, and definitions in current literature are inconsistent.^23^ Data in this study were collected before the pandemic, ensuring that the study population is free from patients suffering from post-COVID-19 condition.

We found less than one-sixth of the differences in ambulatory heart rate measurements to be explained by risk factors for cardiovascular disease, so most of the interindividual differences in heart rate remain unexplained. Even so, markers for poor health (such as inactivity, smoking, higher BMI and lower lung function) were associated with a higher heart rate. Somewhat surprisingly, worse kidney function (ie, lower eGFR) was associated with a lower mean and minimum heart rate. One previous study has shown that lower eGFR is associated with chronotropic incompetence in patients with heart failure with preserved ejection fraction.^24^

We also found lower haemoglobin levels to be associated with lower heart rate, which might seem contraintuitive, since lower haemoglobin physiologically should lead to a compensatory increase in heart rate. This association could perhaps be explained by unmeasured confounding (for example, by dehydration, which can cause both an increase in heart rate and a higher level of haemoglobin), an indication that there could be other unknown determinants of heart rate that our relatively extensive model does not include.

Causal pathways between heart rate and cardiovascular disease have been suggested,^25–27^ but in most studies, heart rate is associated with a similar risk increase for all-cause mortality, cancer mortality and cardiovascular mortality,^1^ which could indicate that higher heart rates are not causally related to cardiovascular disease, but rather markers of poor health. On the other hand, a genome-wide association study has identified heart rate-associated genes which explain 2.5% of the differences in resting heart rate and shown these genes to be associated with increased all-cause mortality,^28^ implying that a causal component to the association between heart rate and mortality may exist. Heart rate only slightly increases the risk of stroke,^6^ which is likely due to the fact that the association between low heart rate and atrial fibrillation is in the opposite direction from the association between heart rates and cardiovascular disease^12^—two major stroke risk factors with different mechanisms.

### Limitations

We have used a large population-based study with extensive information on comorbidities and lifestyle to describe reference ranges and predictors for ambulatory measures of heart rate in the general population. Some limitations exist that need to be considered. The cross-sectional nature of this study does not allow any estimation of the predictive value of different ambulatory heart rate ranges, and though we found large interindividual differences in heart rates even among otherwise healthy individuals, we are not able to study whether ambulatory heart rates predict or cause incident disease. There are also some potential sources of measurement error. We adjusted for habitual leisure time physical activity, but mean heart rate is reasonably also affected by activity during the registration, which we did not adjust for. The mean monitor duration was 24.2 hours with an SD of 1.3 hours. Since the device was both attached and removed when awake, individuals with longer recording duration than 24 hours would have slightly lower sleep/awake time quotas, resulting in somewhat higher mean heart rates. Daytime and night-time hearts rates are reported at fixed times of day and not equivalent to awake/sleeping heart rates. Assuming that subjects did not sleep the full duration of time between 22:00 and 06:00, sleeping heart rates may be somewhat lower than the reported night-time heart rates and the awake heart rate perhaps somewhat higher than the reported daytime heart rate. We lacked data on thyroid hormone levels. This may have reduced the degree to which the differences of the heart rates are explained by the model somewhat. Inclusion of individuals with abnormal thyroid hormone levels could also possibly have resulted in slightly wider reference ranges. The women in the study are mostly post-menopausal, and thus the results may not be generalisable to a pre-menopausal population where the menstrual cycle may also have an impact on ambulatory heart rates. Finally, the most significant limitation of this study is that we have only been able to include individuals between 50 and 65 years of age.

## Conclusions

Normal limits of ambulatory heart rate have a wide range in a healthy middle-aged population sample, and measures of ambulatory heart rate are only explained by clinical correlates to a relatively small degree. The reference ranges presented here can be used in the interpretation of ambulatory ECGs, in particular to determine whether patients with a perception of tachycardia have abnormally elevated mean heart rates.

## Data Availability

Data are available upon reasonable request. Any request for data will need to be approved by the study authors as well as the SCAPIS leadership.
