# Supplementary material for: Reference ranges for ambulatory heart rate measurements in a middle-aged population
Source: Heart. 2024 Apr 5;110(12):831–7. doi: 10.1136/heartjnl-2023-323681 (PMC11137473; doi:10.1136/heartjnl-2023-323681)

Supplementary material

Supplemental table 1. Maximum heart rate during 1 minute in the healthy reference sample

	**Maximum heart rate (beats/min)**	
	Men	Women
Mean (SD)	134 (19)	140 (17)
**Percentiles**		
**2.5**	**103**	**110**
5	106	114
25	120	128
**50**	**132**	**138**
75	146	150
95	168	167
**97.5**	**180**	**174**

Supplemental Table 2. Heart rates in the entire study population

	Mean heart rate (beats/min)		Minimum heart rate$^{\Psi}$ (beats/min)		Average daytime heart rate (beats/min)		Average night-time heart rate (beats/min)	
	Men	Women	Men	Women	Men	Women	Men	Women
**Entire study population (2,755 men and 3,055 women)**								
Mean (SD)	74 (9)	76 (8)	50 (7)	51 (7)	78 (10)	81 (9)	64 (9)	66 (8)
**Percentiles**								
**2.5**	**57**	**61**	**36**	**39**	**59**	**64**	**48**	**51**
5	59	63	38	41	63	67	50	53
25	67	71	44	47	71	75	57	60
**50**	**74**	**76**	**49**	**51**	**78**	**81**	**63**	**65**
75	80	82	53	56	85	87	70	71
95	89	89	62	63	94	95	81	81
**97.5**	**92**	**93**	**65**	**66**	**97**	**98**	**84**	**85**

Supplemental table 3. Multivariable linear regression models for mean and minimum heart rate both as standard linear regression and with linear regression analysis robust to heteroskedasticity

	Mean heart rate	Mean heart rate*	Minimum heart rate	Minimum heart rate*
**Age (per 1 year)**	-0.0 [-0.1,0.0]	-0.0 [-0.1,0.0]	0.1 [0.0,0.1]	0.1 [0.0,0.1]
**Men (vs women)**	-2.8 [-3.5,-2.0]	-2.8 [-3.5,-2.0]	-2.2 [-2.9,-1.6]	-2.2 [-2.9,-1.6]
**Height (per 10 cm)**	-0.8 [-1.2,-0.5]	-0.8 [-1.2,-0.5]	-0.8 [-1.0,-0.5]	-0.8 [-1.0,-0.5]
**Body mass index, kg/m^2**				
**<25**	ref	ref	ref	ref
**25-30**	0.9 [0.4,1.4]	0.9 [0.4,1.4]	0.8 [0.4,1.3]	0.8 [0.4,1.3]
**>30**	1.9 [1.2,2.6]	1.9 [1.2,2.5]	1.4 [0.9,2.0]	1.4 [0.9,2.0]
**Smoking**				
**-never**	ref	ref	ref	ref
**-former**	1.2 [0.7,1.7]	1.2 [0.7,1.7]	0.9 [0.5,1.3]	0.9 [0.5,1.3]
**-current**	2.7 [1.9,3.4]	2.7 [1.9,3.5]	2.2 [1.6,2.9]	2.2 [1.5,2.9]
**High physical activity (vs low)**	-3.6 [-4.1,-3.1]	-3.6 [-4.1,-3.1]	-2.7 [-3.1,-2.3]	-2.7 [-3.1,-2.3]
**Alcohol intake, above median (vs below median)**	0.7 [0.2,1.1]	0.7 [0.2,1.1]	0.8 [0.4,1.2]	0.8 [0.4,1.2]
**Diabetes (yes vs no)**	2.0 [1.1,2.8]	2.0 [1.0,2.9]	2.1 [1.4,2.8]	2.1 [1.3,3.0]
**Hypertension (yes vs no)**	0.4 [-0.1,1.0]	0.4 [-0.2,1.1]	0.1 [-0.4,0.6]	0.1 [-0.4,0.6]
**Using oral betablockers (yes vs no)**	-4.5 [-5.5,-3.4]	-4.5 [-5.6,-3.3]	-0.9 [-1.8,-0.1]	-0.9 [-1.9,-0.0]
**FEV1%predicted, per 10 % increase**	-0.3 [-0.4,-0.1]	-0.3 [-0.4,-0.1]	-0.3 [-0.5,-0.2]	-0.3 [-0.5,-0.2]
**Coronary artery calcium score**				
**0**	ref	ref	ref	ref
**1-99**	0.4 [-0.1,1.0]	0.4 [-0.1,1.0]	0.4 [-0.1,0.8]	0.4 [-0.1,0.8]
**≥100**	0.6 [-0.2,1.4]	0.6 [-0.3,1.4]	0.5 [-0.1,1.2]	0.5 [-0.2,1.2]
**eGFR (per 10 mL/min/1.73m^2)**	0.6 [0.4,0.8]	0.6 [0.4,0.8]	0.4 [0.3,0.6]	0.4 [0.2,0.6]
**Hemoglobin (per 10 g/L increase)**	0.7 [0.5,1.0]	0.7 [0.5,1.0]	0.3 [0.1,0.5]	0.3 [0.0,0.5]
**Constant***	76.1 [75.4,76.8]	76.1 [75.4,76.8]	49.9 [49.3,50.5]	49.9 [49.3,50.5]
Observations	5021	5021	5021	5021
Unadjusted R^2	0.149	0.149	0.143	0.143
Adjusted R^2	0.146	0.146	0.140	0.140

95% confidence intervals in brackets
*robust sandwich variance estimator

Supplemental figure 1. Average day- and night-time heart rates in the healthy reference sample, histogram and sex specific cumulative distribution curves

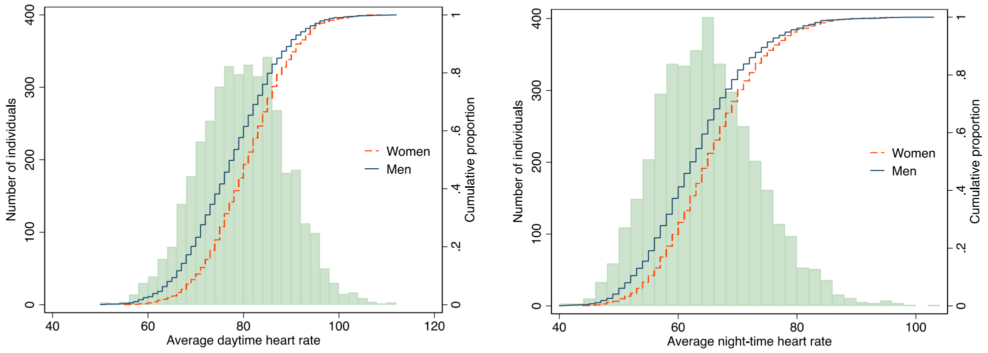

Supplementary figure 2. Mean heart rate, residuals vs fitted values from the multivariable linear regression

2a. Men and women

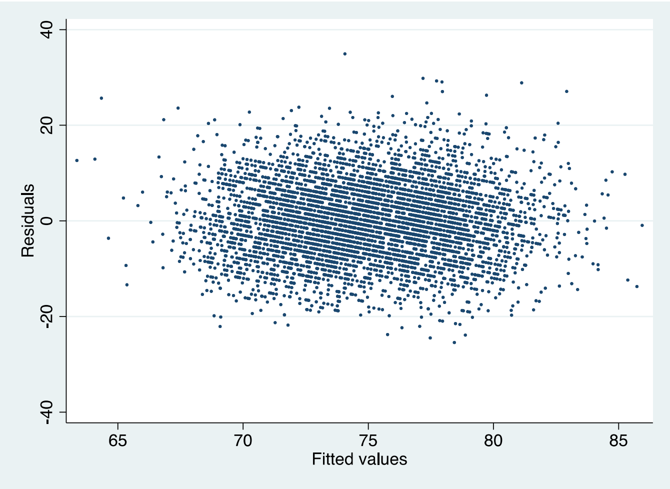

2b Women

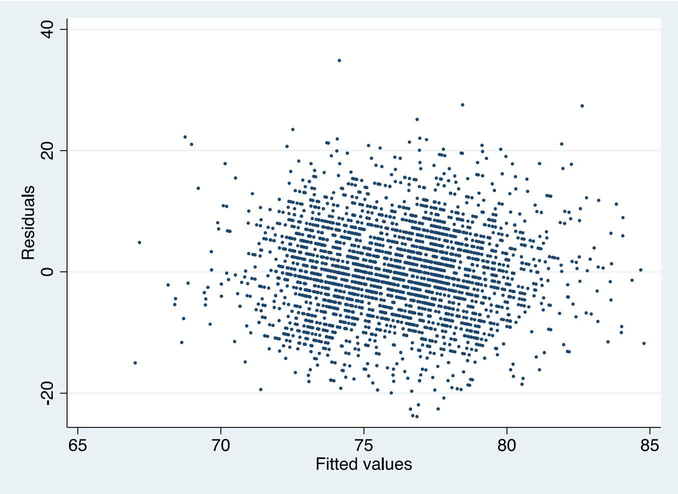

2c. Men

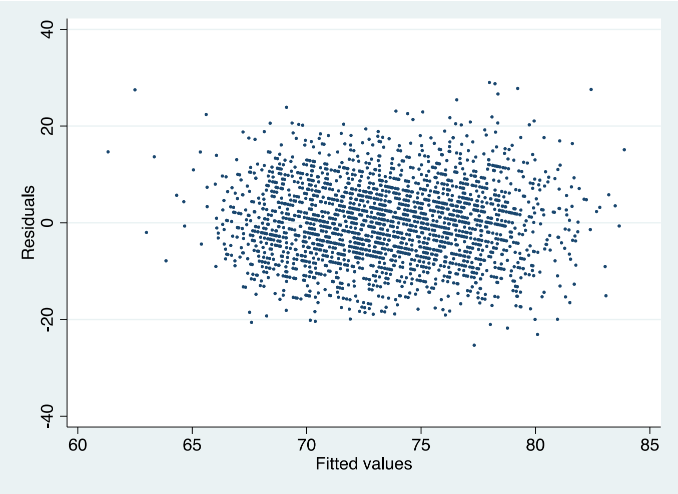

Supplemental figure 3. Histograms of standardized residuals from the mean heart rate multivariable linear regression

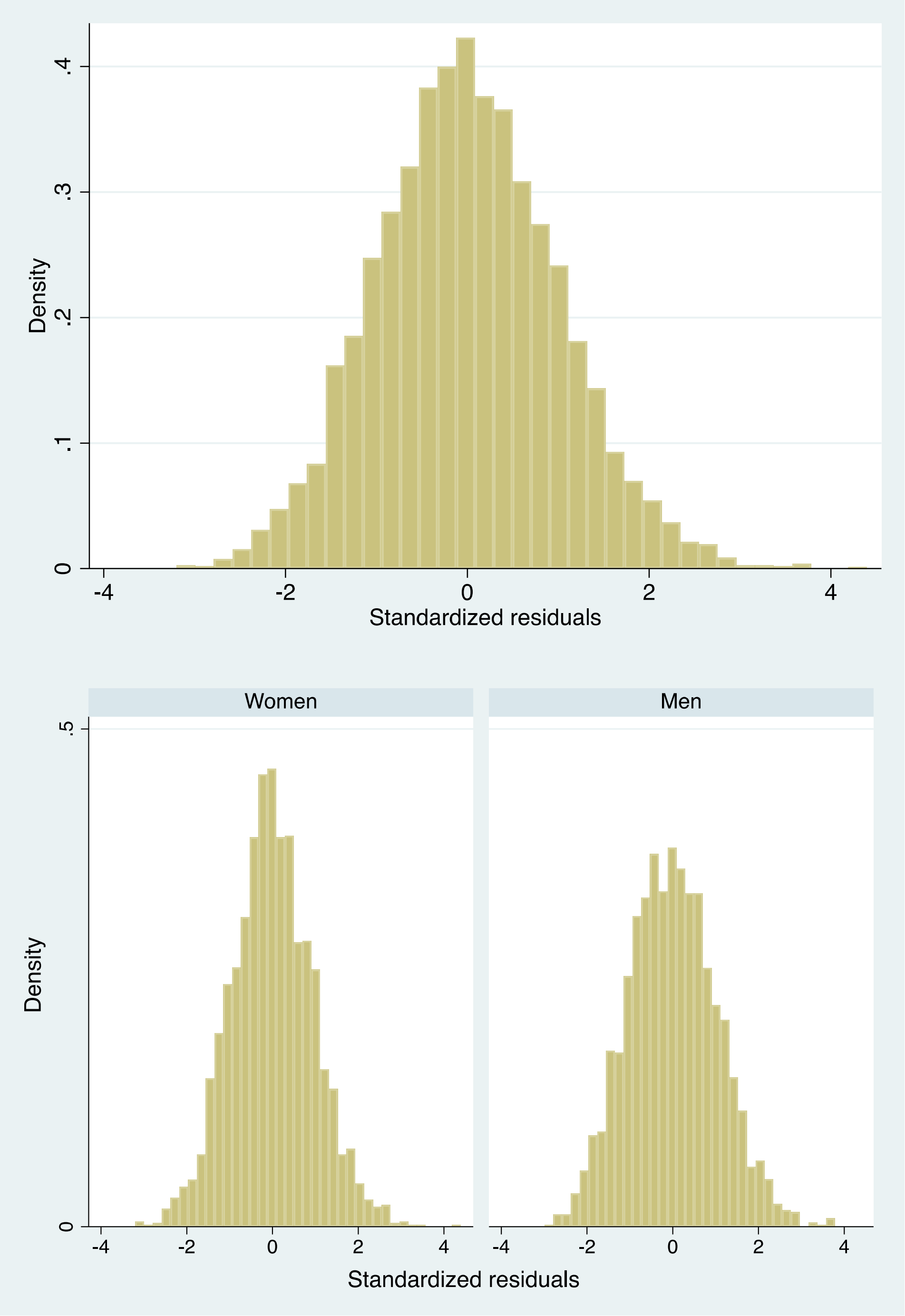

Supplemental figure 4. Minimum heart rate, residuals vs fitted values from the multivariable linear regression

4a. Men and women

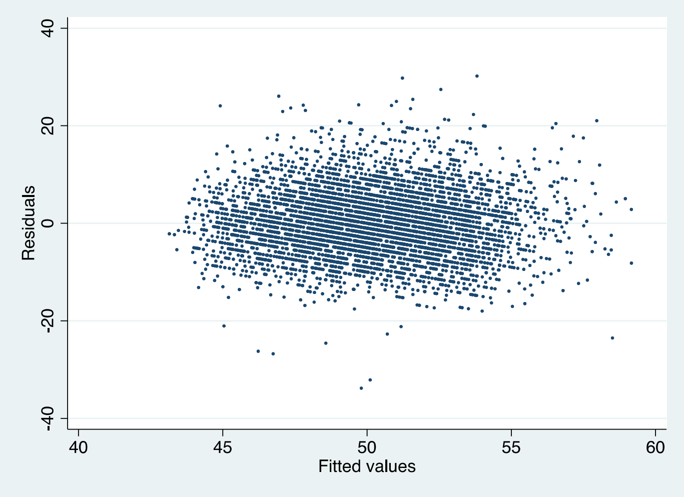

4b. Women

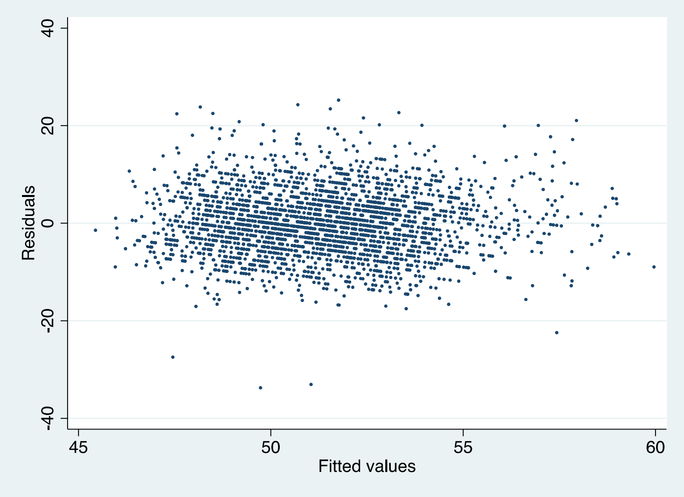

4c Men

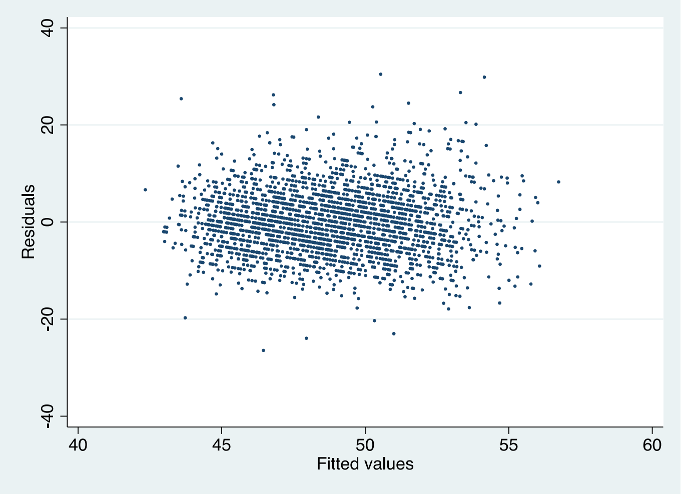

Supplemental figure 5. Histograms of standardized residuals from the multivariable linear regression with minimum heart rate as dependent variable

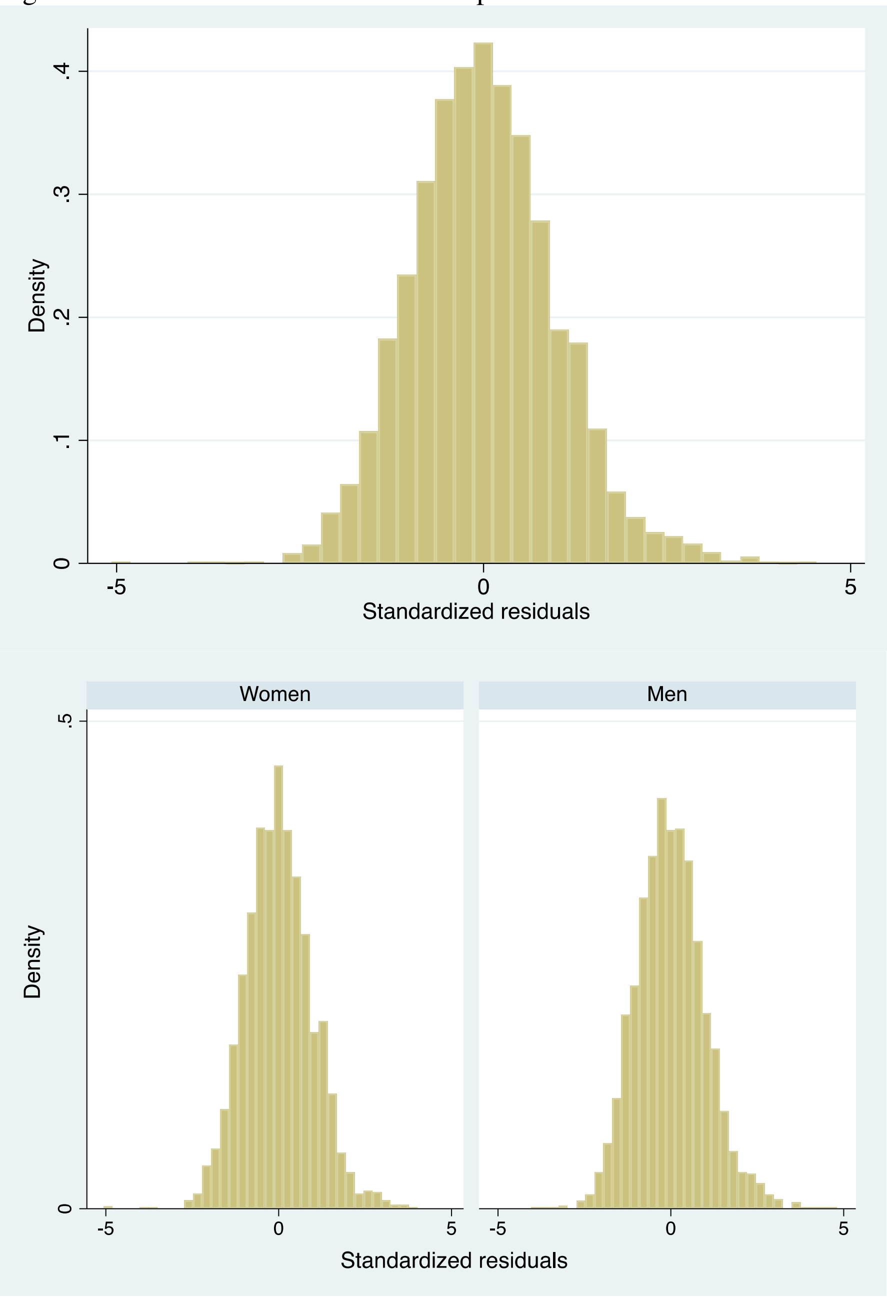

Supplement: Supplementary data [file heartjnl-2023-323681supp001.pdf]
